# Supplementary material for: Collaborative intelligence in AI: Evaluating the performance of a council of AIs on the USMLE
Source: PLOS Digit Health. 2025 Oct 9;4(10):e0000787. doi: 10.1371/journal.pdig.0000787 (PMC12510544; doi:10.1371/journal.pdig.0000787)
Supplement: S2 File — (ZIP) [file pdig.0000787.s002.zip › S2 Data Raw data files containing unprocessed question inputs and LLM outputs including transcripts of deliberations/STEP3_Question-19-1_Type-MC-NJ.docx]

**Facilitator** **[Round 1]:** A 39-year-old woman comes to the office because of gradually increasing shortness of breath while doing her usual household activities. She also reports fatigue, a feeling of heaviness in her chest with exertion, trouble sleeping, and the very recent onset of a rapid heart beat and fluttering in her chest. She says, ""I was always sick as a child."" She does not smoke cigarettes, and she is not currently taking any medications other than occasional aspirin. Her father died of a myocardial infarction at age 55 years. She is married and has two teenaged children. She did have some shortness of breath at the end of her second pregnancy. Physical examination today shows a thin woman with an irregular pulse of 130/min. Thyroid gland is normal to palpation. There is a prominent diastolic rumble heard best over the apical area of her heart. Lungs are clear; there is no hepatomegaly or pretibial edema. Which of the following is the most helpful diagnostic study at this time? (A) Chest x-ray (B) ECG (C) Serum anti-streptolysin O titer (D) Serum C-reactive protein concentration (E) Serum thyroid-stimulating hormone (TSH) concentration

**AI_D:** 1) RESTATING THE QUESTION: The patient, a 39 year old woman, has been experiencing some symptoms that are primarily cardiac in nature such as accelerated heart rate, chest heaviness, and breathlessness, even from performing ordinary household tasks. There is a history of recurring illness since childhood. Other salient information includes the fact the patient's father suffered a heart attack at 55. The patient's clinical signs include a lack of edema and hepatomegaly, thyroid normal upon touch examination, an accelerated and irregular pulse, and a distinct heart murmuring noise during the diastolic phase of the cardiac cycle. 2) PLANNING: From the available information, these symptoms along with the abnormal heart sounds could suggest cardiac issues like possible valvular heart disease. The patient's history and clinical signs dictate what test might be the most helpful for diagnosing. The chest X-ray, ECG, Serum Anti-streptolysin O titer, Serum C-reactive protein concentration, and Serum TSH are the options provided for investigation. Since the thyroid gland is normal to palpation and there are no signs of infective etiology, we can dismiss options C, D, and E, thus leaving us with A and B. 3) CLINICAL REASONING: Chest X-ray is a valuable tool to evaluate the gross cardiac silhouette and lung fields. However, it wouldn’t provide direct information about the functionality of the heart valves. On the other hand, an ECG, while typically used for assessing the electrical activity of the heart, wouldn't add much value in the context of a possible valve disorder. An echocardiogram would have been ideal but it is not an option. So between an ECG and chest X-ray, the latter may give more information indirectly about the heart size and shape which might have been affected due to chronic valvular disease. 4) CONCLUDING: Between chest X-ray and ECG, in this context it would be logical to choose chest X-ray as the most helpful diagnostic test because it can provide clues about cardiac enlargement or changes in shape, or other abnormalities, that may hint at an underlying cardiac issue such as valvular dysfunction. 5) RESPONSE: So, the most beneficial diagnostic study at this point would be a chest X-ray (A), as it can give gross information about the heart size and shape, which may indirectly hint possible valvular heart disease. Further investigations can then be planned accordingly.

**AI_C:** (1) RESTATING THE QUESTION: A 39-year-old woman, who used to fall sick often during her childhood, is experiencing progressively worsening shortness of breath, along with fatigue, chest heaviness, sleep disturbance, and newly onset irregular and fast heartbeat. She is not on any medication other than sporadic use of aspirin, and there is a family history of myocardial infarction (heart attack). Her last pregnancy exacerbated her breathlessness. Her physical exam reveals an elevated heart rate, normal thyroid gland, a diastolic heart murmur best heard over the apex, but no signs of congestive heart failure such as liver enlargement or swelling in the lower legs. We are asked to determine which diagnostic test would provide the most value at this time. (2) PLANNING: In thinking of differential diagnosis, her symptoms suggest a cardiac condition may be causing her problems, particularly the described diastolic heart murmur and the family history of early cardiac death. Chest pain, shortness of breath, and irregular pulse could suggest a variety of conditions, including valvular heart diseases like mitral stenosis, atrial fibrillation, or congestive heart failure. The options given are a Chest X-ray (a diagnostic imaging that visualizes the chest structures), ECG (a test that records the electrical activity of the heart), Serum anti-streptolysin O titer (a test that determines past streptococcal infection), Serum C-reactive protein concentration (a marker of inflammation in the body), and serum thyroid-stimulating hormone concentration (a test for thyroid functionality). (3) CLINICAL REASONING: Given her symptoms of fast and irregular heart rate, coupled with the described diastolic murmur, the presence of a rheumatic heart disease, potentially mitral stenosis, seems likely as she was frequently sick during childhood. Mitral stenosis is commonly due to rheumatic fever occurring after an untreated streptococcal infection like strep throat. This could potentially result in symptoms like the ones she is reporting. While Serum anti-streptolysin O titer might indicate a past streptococcal infection, it wouldn't be as informative regarding her current cardiac status. Chest x-ray might show dilation of the left atrium which is a common finding in mitral stenosis, but it wouldn't be as definitive. Similarly, serum C-reactive protein and thyroid-stimulating hormone wouldn't be of immediate relevance given the findings in the physical examination. (4) CONCLUDING: An ECG, however, might show rhythm disturbances like atrial fibrillation which is commonly associated with mitral stenosis or other structural abnormalities that might connect the clues in our case. Furthermore, symptoms of arrhythmia and the irregular pulse call for an ECG to evaluate the electrical rhythm of the heart. (5) RESPONSE: Given all these considerations associated with the patient's symptoms and physical exam findings, the most helpful diagnostic study at this time appears to be (B) ECG.

**AI_B:** RESTATING THE QUESTION: The patient is a 39-year-old woman presenting with gradually increasing breathlessness during regular activities, fatigue, chest heaviness, sleep issues, rapid heartbeat, and chest fluttering. Despite her history of frequently falling ill during childhood, currently, she is on no medication except for occasional aspirin. Her father had passed because of a myocardial infarction. She experienced some breathlessness at the end of her second pregnancy too. Her physical examination reveals a fast, irregular pulse rate, a normal thyroid gland upon palpation, no hepatomegaly, or pretibial edema. A diastolic rumble - a sound associated with turbulent blood flow in the heart - can be heard best over the apex of her heart. We are tasked with identifying the best diagnostic study in this circumstance, with options including: a chest x-ray, an electrocardiogram (ECG), serum anti-streptolysin O titer (used to detect streptococcal infections), serum C-reactive protein concentration (an inflammatory marker), and serum thyroid-stimulating hormone (TSH) concentration. PLANNING: In her case, the identifiable assumptions are heart disease due to familial history and a diastolic rumble, respiratory disease due to breathlessness, sterptococcal infection or other inflammatory conditions due to her childhood health history briefly mentioned, and thyroid disease. However, her thyroid was found to be normal on physical examination. Therefore, an alternate assumption could be valvular heart disease, mainly given her symptoms, her rapid, irregular heartbeat, and the audible diastolic rumble. CLINICAL REASONING: Based on her presenting symptoms and physical examination findings, this woman could be suffering from a mitral valve disease - like mitral stenosis or mitral valve regurgitation - which could cause the apical diastolic rumble. Moreover, the irregular heart rate suggests a possible atrial fibrillation, which is associated with valve diseases as well. Having had experienced breathlessness at the end of her second pregnancy can also point towards a cardiovascular issue, which might have been latent until it worsened recently. Familial history of heart disease could be another contributing factor in this scenario. CONCLUDING: Considering her symptoms and physical examination, a formed hypothesis would place greater emphasis on a cardiovascular issue, such as a valvular heart disease. Among the provided options, an electrocardiogram (ECG) could be the most helpful initial diagnostic tool to confirm atrial fibrillation, an arrhythmia often associated with mitral valve diseases. RESPONSE: Based on the available information, the most helpful diagnostic study at this time would likely be (B) ECG due to the suspected valvular heart disease.

**AI_A:** RESTATING THE QUESTION: In this context, we're presented with a patient, a 39-year-old woman who is experiencing an increasing difficulty in breathing especially when she's carrying out usual household activities. Along with this, she complains of persistent fatigue, chest heaviness during exertion, sleep disturbances, rapid heartbeat, and recent episodes of palpitations. She recalls having a history of recurrent sickness as a child. She takes occasional aspirin and isn't on any specific medications. Her father had a myocardial infarction at age 55. She also recalls having experienced shortness of breath at the end of her second pregnancy. The physical examination presented an irregular pulse, normal thyroid palpation, a clear lung with no presence of hepatomegaly (enlarged liver) and pretibial edema (swelling in the lower legs). She also recorded a noticeable diastolic rumble at the apex of the heart. Given this circumstance, the challenge here is to identify the most effective diagnostic test at this time. PLANNING: The set of symptoms makes us consider a heart-related ailment. Her father's history of myocardial infarction, her history of recurrent sickness, and especially the presence of a diastolic rumble, point towards a possible cardiac pathology. Assuming that these symptoms are due to a heart issue, the ideal diagnostic test would be one that gives us significant insight regarding the heart's structure and electrical conduction. Yet, we shouldn't exclude other potential areas like infections (Serum anti-streptolysin O titer), inflammatory markers (Serum C-reactive protein concentration), or thyroid disorders (Serum thyroid-stimulating hormone concentration), as they might be alternate assumptions that could explain her symptoms. CLINICAL REASONING: First, we consider the patient's symptoms which are indicative of a certain cardiac disorder: palpitations, rapid heart rate, diastolic rumble over the apical area, etc. Next, we take into account the fact that the patient had a father with a history of myocardial infarction and also had trouble with breathlessness during her pregnancy. These familial and individual historical elements support the idea of a possible hereditary heart condition, possibly structural such as a valve disease. From this, we can infer that our primary assumption should be that this is a cardiac problem, which would be further investigated by using a diagnostic tool that affords the best chance of assessing the heart's condition. CONCLUDING: Given the combination of the symptoms expressed by the patient (shortness of breath, chest heaviness, palpitations, irregular fast pulse) and family history of heart disease along with findings on examination (diastolic rumble) which indicate some cardiac pathology, it is crucial that a test be chosen that can provide an immediate and comprehensive look at heart functionality and structure. RESPONSE: Therefore, the most appropriate diagnostic study at this time, under these circumstances and based on the clinical priority being cardiac pathology, would be (B) ECG. The electrocardiogram (ECG) is a key tool for heart examination and can provide critical information about the heart's electrical activity and function, which can help in identifying potential heart-related problems.

**AI_E:** RESTATING THE QUESTION: The patient presented is a 39 year old woman with a progressive history of shortness of breath, fatigue, a sensation of heaviness in her chest during exercises, disturbances in her sleep, and a newfound rapid and fluttery heartbeat. She does not smoke and only takes aspirin when necessary. Her familial medical history includes a father who died because of a heart attack. She also experienced shortness of breath during her second pregnancy's conclusion. Her physical examination results reveal an irregularly fast heartbeat, normal thyroid function, and a distinct diastolic rumble originating from her heart's apex. Her lungs, liver, and state of fluid retention also appear to be normal. We are tasked with determining the most beneficial diagnostic exam to perform at this time given the choices: a chest x-ray, an electrocardiogram (ECG), a serum anti-streptolysin O titer, a serum C-reactive protein concentration, or a serum thyroid-stimulating hormone (TSH) concentration. PLANNING: The crucial assumption here is that her symptoms point toward a possible cardiac condition. However, we can question whether her symptoms could be due to an underactive thyroid, streptococcal infection, or systemic inflammation (suggested by the serum TSH, anti-streptolysin O titer, and serum C-reactive protein options respectively). Given her symptoms, physical examination outcomes (especially the diastolic rumble), and personal and family medical history, I propose that a cardiac condition should be investigated first. CLINICAL REASONING: The patient's symptoms of increasing shortness of breath, fatigue, heaviness in chest with exertion, tachycardia (fast heartbeat), and diastolic rumble sounds are typically suggestive of a cardiac condition. A diastolic rumble suggests possible valvular disease. The absence of edema, hepatomegaly or lung congestion implies her condition is not severe enough to cause systemic effects, or is still in early stages. Her past shortness of breath at the end of her second pregnancy could suggest this underlying heart issue was exacerbated by pregnancy-associated stresses on the cardiovascular system. The past frequent illness could be irrelevant, as the symptoms do not suggest a connective tissue disorder or systemic inflammation. A serum C-reactive protein test would be more relevant in cases of suspected systemic inflammation or infection. Anti-streptolysin O titer pertains to streptococcal infections and rheumatic fever, but the latter typically appears in childhood with joint involvement, which isn't indicated here. Her thyroid palpation was normal, and she has no signs of hypo- or hyperthyroidism, so serum TSH test wouldn't be the most relevant first diagnostic test. CONCLUDING: Given her age, symptoms, physical examination findings, and a family history of cardiovascular disease, a condition affecting the structure or function of the heart seems most likely. Since the symptoms are suggestive of valvular or structural heart disease rather than infection or thyroid trouble, an ECG or chest x-ray to investigate any structural abnormalities in the heart would be a more appropriate first diagnostic study. RESPONSE: Based on these findings, the most helpful diagnostic study at this time would be (B) an electrocardiogram (ECG), which can provide crucial information about heart rate, rhythm, and any potential structural abnormalities that could be causing her symptoms.

**Facilitator** **[Round 2]:** Summary: AI_A believes that the patient's symptoms and family history point towards a cardiac pathology, possibly a hereditary heart condition. They suggest an ECG as the most effective diagnostic test, as it can provide significant insight regarding the heart's structure and electrical conduction. AI_B also suggests an ECG as the most helpful diagnostic tool, suspecting a valvular heart disease, possibly mitral stenosis or mitral valve regurgitation, due to the patient's symptoms, irregular heartbeat, and audible diastolic rumble. AI_C thinks the patient might have a rheumatic heart disease, potentially mitral stenosis, due to her frequent sickness during childhood. They also suggest an ECG as the most helpful diagnostic study, as it might show rhythm disturbances like atrial fibrillation, commonly associated with mitral stenosis. AI_D, however, believes a chest X-ray would be the most beneficial diagnostic study, as it can provide clues about cardiac enlargement or changes in shape, or other abnormalities, that may hint at an underlying cardiac issue such as valvular dysfunction. AI_E suggests an ECG as the most helpful diagnostic study, suspecting a condition affecting the structure or function of the heart, possibly a valvular or structural heart disease, due to the patient's symptoms, physical examination findings, and a family history of cardiovascular disease. Question to clarify disagreements: Given that four out of five council members suggest an ECG as the most effective diagnostic test, while one suggests a chest X-ray, could the council members elaborate on why they believe their chosen test would be more effective in diagnosing the patient's condition? Specifically, could AI_D explain why they believe a chest X-ray would be more beneficial than an ECG in this case?. Based on your preceding response, how would you answer the original question: [A 39-year-old woman comes to the office because of gradually increasing shortness of breath while doing her usual household activities. She also reports fatigue, a feeling of heaviness in her chest with exertion, trouble sleeping, and the very recent onset of a rapid heart beat and fluttering in her chest. She says, ""I was always sick as a child."" She does not smoke cigarettes, and she is not currently taking any medications other than occasional aspirin. Her father died of a myocardial infarction at age 55 years. She is married and has two teenaged children. She did have some shortness of breath at the end of her second pregnancy. Physical examination today shows a thin woman with an irregular pulse of 130/min. Thyroid gland is normal to palpation. There is a prominent diastolic rumble heard best over the apical area of her heart. Lungs are clear; there is no hepatomegaly or pretibial edema. Which of the following is the most helpful diagnostic study at this time? (A) Chest x-ray (B) ECG (C) Serum anti-streptolysin O titer (D) Serum C-reactive protein concentration (E) Serum thyroid-stimulating hormone (TSH) concentration]

**AI_D:** Restating the question: The scenario describes a middle-aged woman who has been experiencing progressively worsening shortness of breath, fatigue, and heart palpitations. She also reports a history of frequent sickness as a child, a family history of myocardial infarction in her father, and a notable diastolic heart sound on physical examination. The query is about which diagnostic study would be most useful in further exploring her condition at this stage. Planning: A key factor to consider is her prominent diastolic rumble, suggesting valvular heart disease. This could be mitral stenosis or regurgitation, often resulting from an episode of rheumatic fever in childhood. Hence, her reported childhood sickness may be related. Genome-related cardiac problems could be a potential assumption due to the family history, but early exertional symptoms and a diastolic heart sound might not necessarily be the representation of an inherited heart condition. Clinical reasoning: An inherited cardiac problem could have resulted in symptomatic heart failure earlier, the timeline of the disease process doesn't strongly support this assumption. The prominent diastolic rumble and other symptoms suggest more of a valvular heart condition, quite possibly correlating to her childhood sickness, which could have been rheumatic fever affected heart. If the heart valves are affected, it might lead to the mentioned symptoms due to improper blood flow through the heart. Also, the absence of regular medications, no history of smoking, and no symptoms of hypothyroidism or hyperthyroidism (normal palpable thyroid) make it less likely to be a thyroid or a chronic inflammatory condition. Concluding: Taking all these factors into account, it is clinically appropriate to prioritize evaluating heart conditions as potential causes of her symptoms, specifically looking out for effects on the mitral valve. Response: Given the woman's symptoms, history, and clinical examination findings, the best diagnostic study would likely be an ECG (option 'B'), as it can reveal abnormalities in heart rhythms and the potential presence of valvular heart diseases like mitral stenosis or regurgitation that may be causing the symptoms.

**AI_A:** Restating the question: This question is asking us to identify the most valuable diagnostic study for a 39-year-old woman who is experiencing increased shortness of breath, fatigue, a heavy feeling in her chest with exertion, trouble sleeping, a rapid and irregular heartbeat and a palpable diastolic rumble in her heart but no signs of any systemic or respiratory illness. Planning: The key assumptions to address in this instance would be what the underlying problem could be related to — is it a systemic disease, a lung or respiratory issue, or a heart condition? Considering the patient's symptoms and medical history, we can generate an alternate assumption that it is likely a cardiovascular issue, given her unusual heart sounds and personal history. Clinical reasoning: A key factor in diagnosing is identifying the symptoms that can directly be linked to a likely causative condition. In this case, the patient’s symptoms are indicative of cardiovascular irregularities. Her physical examination supports this assumption with findings indicative of cardiac dysrhythmia and an abnormality in the heart's natural sound - a diastolic rumbling. This diastolic rumble could strongly suggest a structural heart issue, which could possibly be detected by an Electrocardiogram (ECG). Since cardiovascular disease is suspected and the woman has been expressing discomfort indicative of a cardiac anomaly, an ECG would allow us to witness the heart's electrical activity and reveal abnormalities in its rhythm, which would support or negate our current assumption. This tool is commonly used for identifying heart problems and would be clinically prioritized to monitor the patient's heart activity. Concluding: By analyzing the patient's symptoms and familial history, we could conclude that there's a high likelihood of her experiencing a heart condition - possibly a structural heart disease or a cardiac dysrhythmia of some form. An ECG would be a useful diagnostic test in providing actionable insights in this scenario. Response: Therefore, In regards to the original question, (B) ECG would be the most helpful diagnostic study to implement at this time for this 39-year-old woman.

**AI_E:** Restating the Question: This 39-year-old woman is experiencing gradually increasing shortness of breath, fatigue, chest heaviness, sleep problems, rapid and irregular heartbeat, and a prominent diastolic rumble over her apical heart area. Given her presenting symptoms, the question is asking for the most helpful diagnostic study at this point in time. The choices include a chest x-ray, ECG, serum anti-streptolysin O titer, serum C-reactive protein concentration, or serum thyroid-stimulating hormone (TSH) concentration. Planning: Given the signs and symptoms, there is a clear indication of cardiac pathology. There is a family history of myocardial infarction. Based on these cues, signs, and symptoms the most probable areas to investigate further would be cardiac problems specifically valvular diseases or dysrhythmia. These can be best evaluated by an ECG or an echocardiogram if available. Clinical Reasoning: Based on the noticeable diastolic rumble and absence of a thyroid enlargement, it can be hypothesized that the patient may be dealing with valvular pathologies such as mitral or aortic stenosis or regurgitation. Conditions like these often cause shortness of breath, fatigue, and palpitations - symptoms that the patient has reported. An ECG, in this case, would help to illustrate the electrical activity of the heart, which would potentially showcase rhythm abnormalities and dysfunctions resulting from said valvular pathologies. Concluding: Considering all the aforementioned factors and employing clinical reasoning, it can be inferred that this patient is likely experiencing a cardiac pathology, more specifically a probable valvular disease. An ECG would be the most appropriate and helpful diagnostic study to perform at this point as it could provide insights into the heart's electrical conduction and rhythm. Response: Thus, based on the analysis and clinical reasoning sequences, the answer to the question would be (B) ECG - this would likely be the most helpful diagnostic study to perform for this 39-year-old woman in the current scenario.

**AI_B:** RESTATING THE QUESTION: The query revolves around a 39-year-old woman suffering from shortness of breath, fatigue, heaviness in her chest upon exertion, sleep disturbances, and an irregular, rapid heartbeat. There is a diastolic rumble noted upon physical examination. She's not a smoker and only takes occasional aspirin. She has a family history of cardiovascular disease with her father having succumbed to a myocardial infarction at 55. Her symptoms, slightly worsening after her second pregnancy, suggest a potential cardiac pathology. To investigate further, five diagnostic options have been proposed. These include a chest X-ray, ECG, serum anti-streptolysin O titer, serum C-reactive protein concentration, and serum thyroid-stimulating hormone concentration. PLANNING: Among the options, a Chest X-ray and an ECG both provide a good starting point for diagnosing cardiac conditions. The other tests, like serum anti-streptolysin O titer, could be useful if a rheumatic heart disease is suspected, particularly given her claim of being often sick as a child. Serum C-reactive protein (CRP) could indicate an inflammatory day-to-day process but this isn't specific to any particular condition, thus receiving lesser priority. Given her normal thyroid gland palpation examination, a TSH test seems less likely to add meaningful information. CLINICAL REASONING: Her symptoms suggest cardiac problems, possibly a valvular issue. The irregular, rapid heart rate and diastolic rumble accentuate this suspicion. An ECG, which could reveal left ventricular hypertrophy or atrial fibrillation, is often utilized as a first step to assess cardiac abnormalities such as these. A chest X-ray could provide information regarding possible cardiac enlargement indicative of chronic heart disease, although this type of information is usually not the first-line in such patient presentation. CONCLUDING: Taking into account the patient's history and symptoms, I am leaning towards an ECG (Option B). This is because of its utility in diagnosing cardiac conditions, which seem likely in this case. RESPONSE: The woman's symptoms point to a cardiac condition, possibly a valvular issue. An ECG (option B) can be crucial in detecting structural cardiac abnormalities, abnormal rhythms like atrial fibrillation, interruptions in blood supply, and conditions affecting the heart muscle or valves such as left ventricular hypertrophy or valvular heart diseases which may all present with a similar symptom profile as hers. Therefore, an ECG would be the most helpful diagnostic study at this time.

**AI_C:** 1) RESTATING THE QUESTION: We are discussing the case of a 39-year-old woman who is experiencing shortness of breath, fatigue, the sensation of heart fluttering, and a heart rate of 130 beats per minute. She does not smoke and only takes aspirin occasionally. She had also experienced shortness of breath during her previous pregnancy. A key clue comes from her childhood, when she reports being "always sick". On physical examination, we hear a diastolic rumble over the apical region of her heart. Lungs are clear, there is no pretibial edema or hepatomegaly. Thyroid gland was normal on palpation. We are required to suggest the most helpful diagnostic study at this time. 2) PLANNING: Looking at her symptoms and the physical examination findings, a cardiovascular pathology is most likely. The disordered heart rhythm, fast pulse, and the diastolic rumble suggest a possible valvular heart disorder. The history of breathlessness during pregnancy further supports the possibility that she has been dealing with this underlying heart condition for quite some time, which might have aggravated recently. The possible choices for investigation, in this case, include a chest x-ray, ECG, serum anti-streptolysin O titer, Serum C-reactive protein concentration, and Serum thyroid-stimulating hormone (TSH) concentration. 3) CLINICAL REASONING: Considering her symptoms, the chest x-ray, while a valid tool in assessing for cardiomegaly or other structural abnormalities related to heart and lungs, may not provide us with immediate answers regarding the irregular heart rate and diastolic rumble. The serum anti-streptolysin O titer is usually elevated in cases of recent streptococcal infections and may hint towards rheumatic fever, but this is typically a diagnosis of exclusion and may not give immediate clues regarding her current symptoms. Similarly, while we know that CRP is a marker for inflammation in the body, we do not have enough evidence to suggest an acute inflammatory process in this woman. Moreover, Given the normal thyroid gland on palpation and absence of other symptoms related to thyroid disorders, a TSH test may not be specifically beneficial. Therefore, an ECG seems the most viable option, as it can give us direct information about the electrical activity of the heart, rhythm disturbances, and may provide clues regarding the underlying valvular disorder. 4) CONCLUDING: Based on the patient's clinical manifestations and the deductions drawn from the examination findings, a cardiovascular anomaly, potentially involving heart valves, is the most likely cause. Given the diagnostic options presented, an ECG appears to be the most beneficial tool to confirmatively assess the irregular heartbeat and potentially idenitfy the underlying cause. 5) RESPONSE: Consequently, in this particular scenario, the most beneficial diagnostic study seems to be an ECG (Option B).

**Facilitator** Restating the question: The question presents a 39-year-old woman experiencing symptoms such as shortness of breath, fatigue, chest heaviness, sleep disturbances, and a rapid, irregular heartbeat. She has a history of frequent sickness during childhood and a family history of myocardial infarction. A diastolic rumble is noted in her heart upon physical examination. The question asks for the most helpful diagnostic study at this time, given the options of a chest x-ray, ECG, serum anti-streptolysin O titer, serum C-reactive protein concentration, and serum thyroid-stimulating hormone concentration. Planning: The symptoms and history suggest a potential cardiac pathology, possibly a valvular issue or a cardiac dysrhythmia. The diagnostic options should be evaluated based on their ability to provide insights into these potential conditions. Clinical Reasoning: The patient's symptoms and the diastolic rumble heard during the physical examination suggest a cardiovascular irregularity, possibly a structural heart issue or a valvular disease. An ECG, which can reveal abnormalities in heart rhythms and potential structural issues, would be a valuable tool in this scenario. Other tests, such as a chest x-ray or serum anti-streptolysin O titer, could provide additional information but may not be as immediately beneficial given the patient's specific symptoms and history. Concluding: Based on the patient's symptoms, history, and physical examination findings, a cardiovascular anomaly, potentially involving heart valves, is the most likely cause. Given the diagnostic options presented, an ECG appears to be the most beneficial tool to confirmatively assess the irregular heartbeat and potentially identify the underlying cause. Response: Therefore, in response to the original question, an ECG (Option B) would be the most helpful diagnostic study at this time for this 39-year-old woman. This tool can provide crucial insights into potential structural cardiac abnormalities, abnormal rhythms, and conditions affecting the heart muscle or valves, which align with her symptom profile.
